# The mechanism of sudden unexpected death in epilepsy: A mini review

**DOI:** 10.3389/fneur.2023.1137182

**Published:** 2023-02-06

**Authors:** Xinyi Sun, Yehui Lv, Jian Lin

**Affiliations:** ^1^School of Basic Medical Sciences, Shanghai University of Medicine and Health Sciences, Shanghai, China; ^2^Institute of Wound Prevention and Treatment, Shanghai University of Medicine and Health Sciences, Shanghai, China; ^3^Chongming Hospital Affiliated to Shanghai University of Medicine and Health Sciences, Shanghai, China

**Keywords:** epilepsy, mortality, sudden unexpected death in epilepsy, mechanism, P-glycoprotein, catecholamines, K^+^ channel, renin-angiotensin system

## Abstract

Sudden unexpected death in epilepsy (SUDEP) is defined as a sudden, unexpected, non-traumatic, non-drowning death in a person with epilepsy. SUDEP is generally considered to result from seizure-related cardiac dysfunction, respiratory depression, autonomic nervous dysfunction, or brain dysfunction. Frequency of generalized tonic clonic seizures (GTCS), prone posture, and refractory epilepsy are considered risk factors. SUDEP has also been associated with inherited cardiac ion channel disease and severe obstructive sleep apnea. Most previous studies of SUDEP mechanisms have focused on cardiac and respiratory dysfunction and imbalance of the neural regulatory system. Cardiac-related mechanisms include reduction in heart rate variability and prolongation of QT interval, which can lead to arrhythmias. Laryngospasm and amygdala activation may cause obstructive and central apnea, respectively. Neural mechanisms include impairment of 5-HT and adenosine neuromodulation. The research to date regarding molecular mechanisms of SUDEP is relatively limited. Most studies have focused on p-glycoprotein, catecholamines, potassium channels, and the renin-angiotensin system, all of which affect cardiac and respiratory function.

## Introduction

Sudden unexpected death in epilepsy (SUDEP) is defined as a sudden, unexpected, non-traumatic, non-drowning death in a person with epilepsy, witnessed or unwitnessed, in which an autopsy does not reveal an anatomical or toxicological cause of death (1). In a large Chinese community cohort of 1,562 epileptic patients, 15 experienced suspected SUDEP during the 5-year follow-up period; SUDEP incidence was 2.34 per 1,000 person-years (2). Sudden epileptic death is believed to be related to cardiac dysfunction, respiratory depression, autonomic nervous dysfunction, and brain dysfunction during seizure; however, the exact mechanism is unclear (3–5). The purpose of this review is to summarize the current knowledge regarding mechanisms of SUDEP.

## SUDEP risk factors

The frequency of generalized tonic-clonic seizures (GTCS) is considered the most important clinical risk factor for SUDEP (3, 6–8): the higher the GTCS frequency, the higher the risk of SUDEP. Data from a pooled analysis of SUDEP risk factors indicate that patients who have one to two GTCS per year are nearly three times as likely to experience SUDEP than patients who do not have GTCS; patients who have more than 50 GTCS per year are more than 14 times as likely to experience SUDEP (8). Prone positioning during seizures is an important risk factor for accidental death. Most instances of SUDEP occur after a generalized seizure; patients are usually found in the prone position (9, 10). In one study, 73.3% of SUDEP patients died in the prone position and prone position was significantly associated with SUDEP (11). Refractory epilepsy is also a risk factor for SUDEP (12). SUDEP accounts for 5–30% of deaths in all epileptic patients and up to 50% of deaths in patients with refractory epilepsy (13). Furthermore, risk of SUDEP is also higher in males, patients who have had epilepsy for many years, patients with ion channel or arrhythmia-related gene mutations, patients with neurological comorbidities, and patients taking multiple antiepileptic agents (8, 14, 15, 17). The occurrence of SUDEP is also associated with genes related to cardiac arrythmia and ion channels, especially the mutation related to long QT syndrome (LQTS), which may increase the risk of sudden death when combined with epilepsy (16–18). Severe obstructive sleep apnea has also been associated with increased risk of SUDEP (19). Seizure incidence is significantly lower in obstructive sleep apnea patients who receive positive airway pressure therapy than patients who are untreated (20). Structural brain damage may also be a SUDEP risk factor. Changes in brain structures and networks involved in central autonomic nerve and respiratory control have been observed in SUDEP patients and those at high-risk for SUDEP (21). These changes are mainly changes in gray matter volume in the hippocampus, amygdala, and thalamus (13, 22–24).

## SUDEP mechanisms

Most studies which have examined the mechanisms underlying SUDEP have focused on cardiac and respiratory dysfunction and imbalance within the neural regulation system.

### Cardiac dysfunction

Epilepsy may induce various transient cardiac effects, including heart rate changes, heart rate variability (HRV), arrhythmia, cardiac arrest and other electrocardiographic abnormalities (25). Acute and adaptive changes in heart rhythm in epileptic patients is one potential pathogenic SUDEP mechanism (18). To some extent, HRV reflects the balance of the sympathetic and parasympathetic autonomic nervous system divisions. An increase in HRV indicates increased parasympathetic activity while a decrease in HRV indicates a relative increase in sympathetic activity (26). In addition, HRV in epileptic patients decreases in the interictal period, especially in patients with temporal lobe epilepsy and drug resistant epilepsy (27). Moreover, reduction in HRV is associated with higher risk of SUDEP (28). Prolongation of the QT interval may be an important cause of ventricular arrhythmias in epileptic patients (29). Chahal et al. (17) reported that a prolonged QT interval in epileptic patients was associated with increased mortality. In particular, long QT syndrome, an inherited cardiac ion channel disease, is characterized by prolonged ventricular repolarization and ventricular arrhythmia, which may cause syncope or sudden cardiac death (30). In a dog model of long QT syndrome, anticonvulsant drugs can trigger torsade de pointes (31), which can progress to ventricular fibrillation and sudden death (32). Repeated seizures may also cause structural changes in the heart, which is another potential SUDEP mechanism. Pansani et al. (33) reported that repeated seizures in rats with epilepsy may damage the function and structure of the heart through regulation of microRNA that leads to myocardial cell hypertrophy and myocardial fibrosis. Similar pathological changes have also been reported in autopsy studies of SUDEP patients (34).

### Respiratory dysfunction

Central and obstructive apnea and respiratory arrest have also been suspected as mechanisms underlying SUDEP. Hypoxemia caused by obstructive laryngospasm and subsequent respiratory arrest may be a mechanism of accidental sudden death in epileptic patients (35, 36). Tavee et al. (37) reported severe laryngospasm, continuous inspiratory wheezing, and cyanosis during a GTCS in a patient with refractory epilepsy. In animal studies, laryngospasm has been associated with seizure-associated reflux of gastric acid into the throat (35, 38) as well as seizure-associated increased recurrent laryngeal nerve discharge (36). Reflux is probably the cause of epilepsy-associated laryngeal spasm. In a rat epilepsy model, ST segment elevation on electrocardiography, intermittent apnea, and electroencephalography narrowing due to hypoxia were observed after gastric reflux entered the throat (38). Another rat study reported that blocking reflux into the esophagus could eliminate sudden epileptic death (35).

Amygdala activation may cause central apnea and sudden death in epileptic patients. Spread of seizure activity to the amygdala induces central apnea and decreased oxygen saturation (39–41). An area in the human amygdala that inhibits respiration and elicits apnea has been identified in children with epilepsy (42). Many patients with epilepsy are completely unaware of their apnea and do not report dyspnea (41). In addition, in a mouse model of SUDEP, electrolytic damage of the amygdala significantly reduced the incidence of seizure-induced respiratory arrest (S-IRA) and death (43). However, other studies have reported that seizures involving the amygdala are not accompanied by apnea/hypoventilation or that apnea/hypoventilation precedes the seizure. These findings indicate that amygdala involvement may not be important for induction of apnea/hypoventilation in all seizures (44).

Pulmonary edema/congestion is the most common pathological lung finding in SUDEP patients (34). In a forensic analysis of nine SUDEP cases, all exhibited pulmonary edema, pulmonary congestion, alveolar hemorrhage, and pulmonary small bronchiole wall contraction (45). GTCS are associated with neurogenic pulmonary edema (NPE). In post-ictal pulmonary edema, GTCS are the most frequently reported type (46). In one post-ictal neurogenic pulmonary edema study, five of 47 patients had symptoms of pulmonary edema and all five had GTCS (47). Moreover, the presence of an abnormality on chest radiography is significantly associated with the duration of the preceding GTCS (48). In animal models of epilepsy, pulmonary vascular pressure increases in proportion to the duration of seizure. This induced hypertension discharges fluid from the vascular compartment into the pulmonary parenchyma, causing pulmonary edema (49). This may be the mechanism of neurogenic pulmonary edema caused by epilepsy.

### Neurotransmitter dysfunction

5-hydroxytryptamine (5-HT) and adenosine may participate in the pathophysiological mechanism of SUDEP (50). 5-HT plays a neuroregulatory role in respiratory control. It provides tension and excitability drive for multiple components of the respiratory network, detects changes in tissue pH/CO_2_, and regulates ventilation by affecting neurotransmitter release (51). In DBA/1 mice, S-IRA is related to a defect in 5-HT neurotransmission in the dorsal raphe nucleus (52). Light stimulation of 5-HT neurons in the dorsal raphe nucleus and use of selective serotonin reuptake inhibitors and the antiepileptic drug fenfluramine can enhance the effect of 5-HT and reduce S-IRA incidence (52–54). 5-HT_3_ and 5-HT_4_ receptors may be involved in the above mechanism (53, 54). In a rat epilepsy model, seizures induced by pilocarpine can cause depletion of 5-HT in the hippocampus and significantly damage serotonergic neurons in the raphe nucleus (55). Adenosine signaling has a variety of beneficial and harmful effects in the context of epilepsy. Inhibition of adenosine, which leads to respiratory dysfunction during seizure, may be an important SUDEP mechanism (56). In DBA/2 epileptic mice, blockade of adenosine metabolism was significantly associated with increased incidence of S-IRA, while adenosine A_2_ receptor antagonists were significantly associated with lower incidence (57). A_1_ receptor activation with specific agonists can inhibit drug-resistant epileptic events in human temporal cortex slices from drug-resistant patients (58). In patients with temporal lobe epilepsy and hippocampal sclerosis, density of cortical A_2A_ receptors was significantly lower in those with a higher risk of SUDEP, which suggests impaired neuroglial dysfunction and adenosine regulation in these patients. In addition, amygdala A_1_ receptor density was increased in the high-risk patients, which may contribute to peri-ictal amygdala dysfunction in SUDEP (59). Adenosine is closely associated with SUDEP and adenosine receptors may play an important role.

## Molecular mechanism

### P-glycoprotein

P-glycoprotein (P-gp) may be involved in SUDEP, which is the main cause of death in patients with refractory epilepsy (60). Multidrug resistance in patients with refractory epilepsy is primarily related to overexpression of ABC transporters such as P-gp (61, 62). Regardless of metabolic biotransformation, the biological distribution of antiseizure medications and their metabolites depends on functional expression of ABC transporters in the blood–brain barrier, intestine, liver, and kidney (61). However, high-frequency uncontrolled seizures can induce expression of ABC transporters such as P-gp in excretory organs and cells which normally do not express them such as neurons and cardiomyocytes; this increases the risk of refractory epilepsy (61). The expression of P-gp in neurons and myocardial cells can significantly reduce the resting membrane potential (−60 to −10 mV) and affect function in a manner that predisposes to epilepsy, malignant arrhythmia, and sudden accidental death (61, 62). Auzmendi et al. (60) reported that repeated induction of seizures in Wistar rats causes P-gp expression, electrocardiography changes, and increased mortality; these findings may be related to depolarization caused by myocardial cell P-gp expression.

### Catecholamines

Status epilepticus causes release of a large amount of catecholamines (63), which can cause myocardial ischemia, calcium ion overload, oxidative stress, and mitochondrial dysfunction that lead to cardiac damage (64). In animal studies, repeated induction of S-IRA in DBA/1 mice can cause ventricular calcification necrosis; the incidence and lesion size depend on the total number of S-IRA episodes (65). Verrier et al. (66) introduced the concept of “epileptic heart,” which is “a heart and coronary vasculature damaged by chronic epilepsy as a result of repeated surges in catecholamines and hypoxemia leading to electrical and mechanical dysfunction.”

### K^+^ channel

Kv1.1 belongs to the Shaker subfamily of voltage-gated potassium channels and is widely expressed throughout the nervous system, serving as a key regulator of neuronal excitability (67, 68). In wild-type mice, Kv1.1 can be detected in brain nuclei associated with heart and lung function including the basolateral amygdala nucleus, dorsal respiratory group nuclei, dorsal motor nucleus of the vagus nerve, nucleus ambiguus, ventral respiratory column nuclei, and the pontine respiratory group nuclei. It is also found in the posterior trapezoidal nucleus and central area nucleus, which are crucial for chemical sensing (69). Neurons in the posterior trapezium nucleus directly regulate respiration in response to CO_2_/hydrogen ion changes in tissues and control respiration by integrating information from several respiratory centers, including the raphe medulla (70). Kv1.1 subunits control spontaneous excitatory synaptic activity of pyramidal neurons in the basolateral amygdala (71). Kcna knockout mice lack the Kv1.1 subunit and are used as a genetic model of SUDEP (72). In these mice, the inhibitory control of interneurons in the central lateral amygdala nucleus is reduced and abnormal parasympathetic transmission leads to impaired neural control of cardiac rhythm (69, 71, 73). With Kv1.1 deficiency, seizures may cause proliferation of glial cells in the nuclei of the heart and lung centers, which may cause abnormal breathing (69). Kv1.1 deficiency also reduces the inhibitory control of interneurons in the central lateral amygdala and the overexcitation related to seizure inhibition (71). In Kv1.1-deficient mice, epileptic seizures cause abnormal parasympathetic nerve transmission, which leads to impaired neural control of heart rhythm and malignant arrhythmias (73).

In animals with chronic epilepsy, levels of the Kv4.2 myocardial voltage-gated potassium channel are decreased (74). The Kv4.2 subunit contributes to the pore-forming region of channels that express a transient A-type potassium ion current in hippocampal CA1 pyramidal cell dendrites. It is the main medium of hyperpolarized A-type current in the brain, plays an important role in signal processing and synaptic integration, and is a key regulator of neuronal excitability (75, 76). Compared with wild-type mice, the latency to seizure and status epilepticus onset is lower in Kv4.2 knockout mice (76). Silencing of Kv4.2 is mediated by miR-324-5p (75, 77). In epileptic mice, increased Kv4.2 mRNA silencing causes decreased Kv4.2 protein level and production of type A current; its role in regulating neuronal excitability is also limited (77). Inhibition of miR-324-5p can reduce the frequency of spontaneous seizures and epileptic spikes between seizures and produce neuroprotective and antiepileptic effects (75, 77).

### Renin-angiotensin system

The renin-angiotensin system has also been implicated in SUDEP (78). Angiotensin II is the main peptide of the system. Various signal pathways in the central nervous system are stimulated by angiotensin II receptor-1 (ATR1) and angiotensin II receptor-2 (ATR2) (79). Activation of ATR1 is pro-inflammatory and pro-epileptogenic (80). Angiotensin converting enzyme and ATR1 are upregulated in the brain of rats with repetitive seizures (81). We speculate that repeated seizures will lead to upregulation of ATR1 in the brain, causing pro-inflammatory and pro-epileptogenic effects that may lead to SUDEP. Other renin-angiotensin system pathways in the nervous system include angiotensin-(1–7) binding to the receptor Mas (82, 83). Angiotensin-(1–7) participates in the learning and memory process that takes place in the central marginal region of the brain. In chronically stimulated epileptic rats, levels of thimet oligopeptidase [the main enzyme involved in generation of angiotensin-(1–7)], angiotensin-(1–7), and receptor Mas transcripts are elevated (82). However, the effect of this on risk of SUDEP is unknown.

### Other mechanisms

Oxygen-conserving reflexes (OCR), amygdala rapid kindling (ARK), and central nervous system damage owing to repeated GTCS may also be potential mechanisms of SUDEP. Biggs et al. (84) reported that in epileptic rats, induction of OCR causes fluctuations in heart rate and respiratory rate similar to human SUDEP. The ability of the carotid body to stimulate respiratory restart appears to be impaired during seizures (85). Totola et al. (86) reported that in epileptic rats, the number of Fos-immunoreactive neurons in the posterior trapezoidal nucleus, raphe magnus nucleus, and nucleus tractus solitarius decreased after ARK; in addition, the ventilatory volume decreased significantly. ARK damages respiratory neurons in the brain stem, resulting in respiratory dysfunction. After a single GTCS, the blood–brain barrier exhibits signs of inflammation, neuronal damage, and transitory destruction (87). Therefore, repeated GCS attacks may cause central nervous system damage and SUDEP.

**Figure 1:**
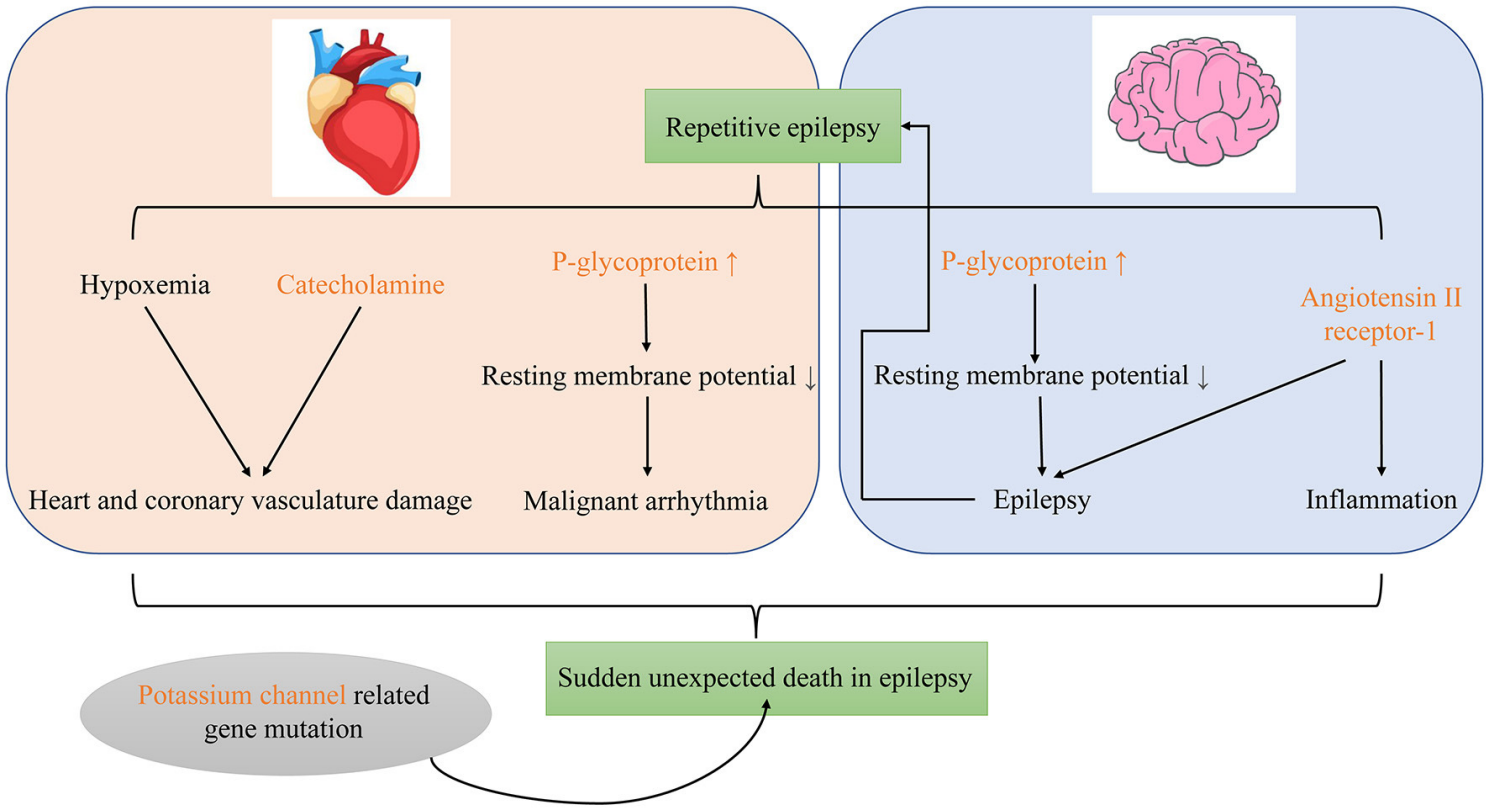
Possible molecular mechanisms of sudden unexpected death in epilepsy.

To sum up, catecholamine, P-gp and ATR1 may participate in SUDEP as important molecular biomarkers. The gene mutation related to potassium channel may also involve in its occurrence (Figure 1).

## Discussion

Among the various SUDEP risk factors, frequency of GTCS appears to be the most important. Prone position, male sex, chronic epilepsy, ion channel or arrhythmia-related gene mutations, neurological comorbidities, polytherapy, long QT syndrome, obstructive sleep apnea, and structural brain damage are other potential factors.

Previous studies of SUDEP mechanisms have focused on malignant arrhythmias, myocardial cell hypertrophy, myocardial fibrosis, central and obstructive apnea, pulmonary edema, and abnormal regulation of the neurotransmitters 5-HT and adenosine. Research of underlying molecular mechanisms has been limited; therefore, SUDEP is often difficult to distinguish from other causes of sudden death (88).

There are many possible molecular mechanisms of SUDEP. First, epileptic seizures induce the expression of P-gp in neurons and myocardial cells, which reduces resting membrane potential and predisposes to development of epilepsy, malignant arrhythmias, and sudden death. Second, after status epilepticus, a large amount of catecholamines are released, which can cause myocardial ischemia, calcium overload, oxidative stress, and mitochondrial dysfunction. In turn, these may cause myocardial damage. Third, abnormal potassium channels may increase the risk of cardiopulmonary dysfunction during seizures. Fourth, repeated seizures lead to upregulation of ATR1 in the brain, causing pro-inflammatory and pro-epileptogenic effects. Finally, OCR, ARK, and central nervous system damage caused by repeated GTCS may be involved as well.

The mechanism of SUDEP is complex and most previous studies have focused on cardiac and respiratory dysfunction and imbalance of the neural regulatory system. Through a systematic literature review, we speculate on the mechanism of certain SUDEP cases as follows: (1) Repeated seizures cause chronic structural damage to the brain, especially the respiratory and cardiac centers. This cumulative damage causes cumulative increased risk of sudden death. (2) During seizures, especially GTCS, sudden neurological disorder and cardiac respiratory dysfunction may cause sudden death. (3) Deletions in ion channel genes deletion increase the risk of cardiopulmonary dysfunction during seizures. Overall, this review summarizes the existing mechanisms and molecular mechanisms of SUDEP, hoping to update the research progress and provide useful reference for forensic scholars in routine cases.

## Author contributions

XS and YL designed the framework of the review and drafted the manuscript. YL and JL provided supervision and contributed to manuscript writing and editing. All authors have read and approved the latest version of the manuscript.
